# Inertial Sensor-Based Two Feet Motion Tracking for Gait Analysis

**DOI:** 10.3390/s130505614

**Published:** 2013-04-29

**Authors:** Tran Nhat Hung, Young Soo Suh

**Affiliations:** Department of Electrical Engineering, University of Ulsan, Namgu, Ulsan 680-749, Korea; E-Mail: hungtn306@gmail.com

**Keywords:** gait analysis, inertial navigation system, inertial sensors, computer vision, Kalman filter

## Abstract

Two feet motion is estimated for gait analysis. An inertial sensor is attached on each shoe and an inertial navigation algorithm is used to estimate the movement of both feet. To correct inter-shoe position error, a camera is installed on the right shoe and infrared LEDs are installed on the left shoe. The proposed system gives key gait analysis parameters such as step length, stride length, foot angle and walking speed. Also it gives three dimensional trajectories of two feet for gait analysis.

## 1. Introduction

Gait analysis is the systematic study of human walking motion [1]. Gait analysis is used to evaluate individuals with conditions affecting their ability to walk. It can be used for health diagnostics or rehabilitation.

There are mainly two kinds of systems for gait analysis: outside observation systems and wearable sensor systems. In outside observation systems, a camera [2], sensors on the floor [3] and optical remote sensors [4] are used to observe walking motion. Advantages of outside observation systems are their high accuracy. The disadvantage is that it requires a dedicated experiment space and the walking range is rather limited.

Various wearable sensors [5] are used for gait analysis, including force sensors [6], goniometer [1] and inertial sensors [7,8]. The main advantage of wearable sensor systems is that it does not require a dedicated space for experiments. Thus gait analysis can be performed during everyday life, where more natural walking can be observed.

Recently inertial sensors have received lots of attention as wearable sensors for gait analysis. There are two types of inertial sensor-based systems. In [7,8], angles of leg joints are estimated, where attitude estimation algorithms are applied using inertial sensor data. In [9], an inertial navigation algorithm [10] is used to estimate a foot movement. By installing inertial sensors on a shoe, foot motion (position, velocity and attitude) can be estimated quantitatively. Many similar systems [11–13] are also developed for personal navigation systems. This paper is closely related to the latter approach, where an inertial navigation algorithm is used to track foot motion.

Inertial navigation algorithm-based foot motion analysis is in most cases [9,11–13] done only for a single foot. However, two feet motion tracking provides more information for the gait analysis. Theoretically, two feet motion tracking can be done by simply attaching an inertial sensor on each shoe instead of on a single shoe. However, inter-shoe distance error diverges as time goes by and the relative position between the left and right foot becomes very large. To maintain the accurate relative position between two feet, it is necessary to measure inter-shoe distance.

In [14], two feet motion is estimated in the context of a personal navigation system. In the system, an inertial sensor is attached on each foot and the distance between two feet is measured using a sonar sensor. Since the system is developed for a personal navigation system, the main interest is the accurate position estimation of a person.

In this paper, we propose inertial-sensor based two feet motion tracking system for gait analysis. An inertial sensor unit is installed on each shoe. The position and attitude between two shoes are estimated using a camera on one shoe and infrared LEDs on the other shoe. Using the proposed system, two feet motion (position, velocity and attitude) can be estimated. We note that only the inter-shoe distance (scalar quantity) is measured in [14]. Thus the relative position between two feet can only be obtained after many walking steps. The fact that the relative position cannot be computed is not a problem in [14] since the goal is to estimate a person's position. On the other hand, the relative position and attitude between two feet can be accurately estimated in the proposed system. Thus the relative location between two feet can be obtained from the first step as long as a camera on the right shoe can see the landmark on the left shoe.

## 2. System Overview

**Figure 1.**
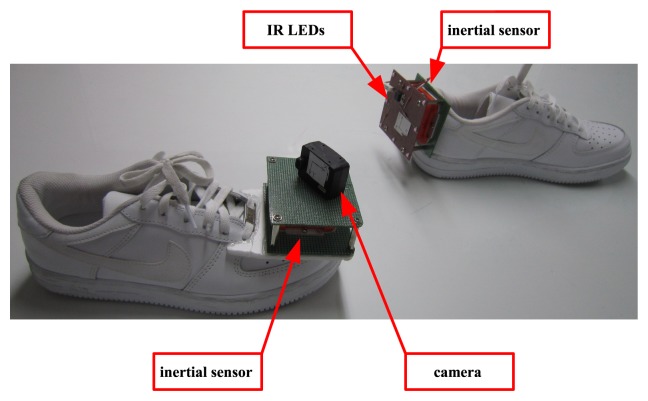
Picture of the proposed system.

The picture of the proposed system is given in Figure 1. Two IMUs (XSens MTi) are attached on both feet. An USB camera (Pointgrey FireFly MV; is attached on the right foot and eight infrared LEDs are attached on the left foot. The movement of two feet is estimated using an inertial navigation algorithm. The relative position between two feet is estimated by capturing the LEDs on the left foot from the camera on the right foot.

As we can see in Figure 1, the sensor unit size is rather large. This may affect the walking patterns. We note that no conscious effort was given to make the system smaller since the purpose of this paper is to demonstrate feasibility of a wearable gait analysis system combining a camera and an inertial sensor unit.

**Figure 2.**
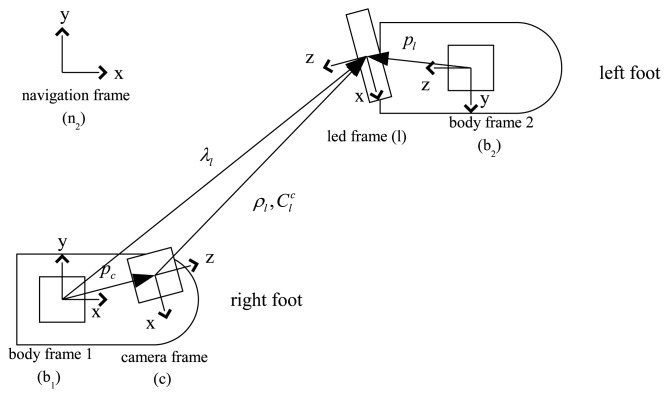
Five coordinate systems (indicated coordinate axes are top-view).

Five coordinate systems are used in the paper (see Figure 2). Three axes of the body 1 (2) coordinate system coincide with three axes of IMU on the right (left) foot. The origin of the camera coordinate system coincides with the pinhole of the camera. The LED coordinate system is defined as in Figure 2. The navigation coordinate system is used as the reference coordinate system. The *z* axis of the navigation coordinate system coincides with the local gravity vector and the *x* axis can be chosen arbitrarily.

A vector *p* ∈ *R*^3^ expressed in the “A” coordinate system is sometimes denoted by [*p*]*_A_* to emphasize that a vector *p* is expressed in the “A” coordinate system. When there are no concerns for confusion, [*p*]*_A_* is just denoted by *p*. A symbol $C_{B}^{A}$ is used to denote the rotation matrix between “A” and “B” coordinate systems. In this paper, symbols *b*_1_, *b*_2_, *n*, *c*, *l* are used to denote body 1, body 2, navigation, camera and LED coordinate systems, respectively.

Let [*r*_1_]*_n_* ∈ R^3^ and [*r*_2_]*_n_* ∈ *R*^3^ be the origins of the body 1 and 2 coordinate systems, respectively. [*r*_1_]*_n_* and [*r*_2_]*_n_* denote the positions of the left and right foot in the navigation coordinate systems. The objective of this paper is to estimate [*r*_1_]*_n_* and [*r*_2_]*_n_*, which are estimated separately using an inertial navigation algorithm. The errors in [*r*_1_]*_n_* and [*r*_2_]*_n_* are compensated by computing [*r*_1_]*_n_* − [*r*_2_]*_n_* using vision. To compute [*r*_1_]*_n_* − [*r*_2_]*_n_*, we introduce some variables in the following.

In Figure 2, [*p_c_*]*_b_*_1_ ∈ *R*^3^ denotes the origin of the camera coordinate system in the body 1 coordinate system and [*p_l_*]*_b_*_2_ denotes the origin of the LED coordinate system in the body 2 coordinate system. Note that *p_c_* and $C_{c}^{b_{1}}$ are constant since the camera and IMU 1 are attached on a shoe. Similarly, *p_l_* and $C_{c}^{b_{2}}$ are also constant.

[*λ_l_*]*_b_*_1_ and [*ρ_l_*]*_c_* denote the origin of the LED coordinate system in the body 1 and camera coordinate systems, respectively. As a person is walking, *λ_l_* and *ρ_l_* are continuously changing. We note that *λ_l_* and *ρ_l_* can be estimated when the camera captures the LED image.

From the vector relationship in Figure 2, we have

(1) $${\left[\lambda_{l}\right]}_{b_{1}}={\left[p_{c}\right]}_{b_{1}}+C_{c}^{b_{1}}\;{\left[\rho_{l}\right]}_{c}$$

The origin of the LED coordinate system can be expressed in the navigation coordinate system as follows:

(2) $${\left[r_{1}\right]}_{n}+C_{b_{1}}^{n}\;{\left[\lambda_{l}\right]}_{b_{1}}={\left[r_{2}\right]}_{n}+C_{b_{2}}^{n}\;{\left[p_{l}\right]}_{b_{2}}$$

Inserting Equation (1) into Equation (2), we have

(3) $${\left[r_{1}\right]}_{n}-{\left[r_{2}\right]}_{n}=C_{b_{2}}^{n}\;{\left[p_{l}\right]}_{b_{2}}-C_{b_{1}}^{n}\;{\left[p_{c}\right]}_{b_{1}}-C_{b_{1}}^{n}C_{c}^{b_{1}}\;{\left[\rho_{l}\right]}_{c}$$

The relationship (3) is used in Section 3.3.

## 3. Motion Estimation Algorithm

In this section, an inertial navigation algorithm to estimate two feet motion is given. In Sections 3.1 and 3.2, a basic inertial navigation algorithm using an indirect Kalman filter is given. In Sections 3.3 and 3.4, measurement equations for the Kalman filter are given. In Section 3.5, an implementation issue of the proposed algorithm is discussed.

### 3.1. Basic Inertial Navigation Algorithm

Let *r̂*_1_ and *r̂*_2_ be estimates of *r*_1_ and *r*_2_. In this paper, we use the inertial navigation algorithm in [9,15]. We only state an algorithm for *r̂*_1_ since the algorithm for *r̂*_2_ is exactly the same.

Let *υ*_1_ ∈ *R*^3^ be the velocity of the right foot and *q*_1_ ∈ *R*^4^ be the quaternion representing the rotation between the navigation and body 1 coordinate system. It is standard [10] that *q*_1_, *r*_1_ and *υ*_1_ satisfy the following:

(4) $$\begin{array}{lll} {\dot{q}}_{1} & = & \frac{1}{2}\Omega (\omega_{b_{1}})q_{1}\\ {\dot{\upsilon }}_{1} & = & a_{b1}\\ \dot{r} & = & \upsilon_{1} \end{array}$$

where *ω_b_*_1_ ∈ *R*^3^ is the angular rates of the body 1 coordinate system with respect to the navigation coordinate system and *a_b_*_1_ is the external acceleration acting on IMU 1. For a vector *ω* = [*ω_x_ ω_z_ ω_y_*]′ ∈ *R*^3^, Ω(*ω*) is defined by

$$\Omega \left(\omega \right)≜\left[\begin{array}{cccc} 0 & -\omega_{x} & -\omega_{y} & -\omega_{z}\\ \omega_{x} & 0 & \omega_{z} & -\omega_{y}\\ \omega_{y} & -\omega_{z} & 0 & \omega_{x}\\ \omega_{z} & \omega_{y} & -\omega_{x} & 0 \end{array}\right]$$

Angular rates *ω_b_*_1_ and external acceleration *a_b_*_1_ are measured using gyroscopes and accelerometers in IMU 1. Let *y_g,_*_1_ ∈ *R*^3^ and *y_a,_*_1_ ∈ *R*^3^ be gyroscope and accelerometer outputs of IMU 1, then *y_g,_*_1_ and *y_a,_*_1_ are given by

(5) $$\begin{array}{lll} y_{g,1} & = & \omega_{b1}+\upsilon_{g,1}+b_{g,1}\\ y_{a,1} & = & C_{n}^{b_{1}}\;\tilde{g}+a_{b_{1}}+\upsilon_{a,1} \end{array}$$

where *g̃* ∈ *R*^3^ is the earth's gravitational vector and *b_g,_*_1_ ∈ *R*^3^ is the gyroscope bias. Measurement noises *υ_g,_*_1_ ∈ *R*^3^ and *υ_a,_*_1_ ∈ *R*^3^ are assumed to be white Gaussian noises whose covariances are given by *R_g,_*_1_ and *R_a,_*_1_, respectively.

Inserting Equation (5) into Equation (4), we obtain the following:

(6) $$\begin{array}{lll} {\dot{\hat{q}}}_{1} & = & \frac{1}{2}\Omega (y_{g,1}-{\hat{b}}_{g,1}){\hat{q}}_{1}\\ {\dot{\hat{\upsilon }}}_{1} & = & {\left(C({\hat{q}}_{1})\right)}^{\prime }y_{a,1}-\tilde{g}\\ {\dot{\hat{r}}}_{1} & = & {\hat{\upsilon }}_{1} \end{array}$$

where *C*(*q*) for a quaternion *q* = [*q*_0_ *q*_1_ *q*_2_ *q*_3_]′ is defined by

(7) $$C\left(q\right)=\left[\begin{array}{ccc} 2q_{0}^{2}+2q_{1}^{2}-1 & 2q_{1}q_{2}+2q_{0}q_{3} & 2q_{1}q_{3}-2q_{0}q_{2}\\ 2q_{1}q_{2}-2q_{0}q_{3} & 2q_{0}^{2}+2q_{2}^{2}-1 & 2q_{2}q_{3}+2q_{0}q_{1}\\ 2q_{1}q_{3}+2q_{0}q_{2} & 2q_{2}q_{3}-2q_{0}q_{1} & 2q_{0}^{2}+2q_{3}^{2}-1 \end{array}\right]$$

Variables for the left foot (*υ*_2_, *q*_2_, *a_b_*_2_, *ω_b_*_2_, *y_g,_*_2_, *y_a,_*_2_, *b_g,_*_2_, *υ_g,_*_2_ and *υ_a,_*_2_) are defined with the same way as in the right foot. *r̂*_2_ can be computed using Equation (6) if the left foot variables are used instead of the right foot variables.

### 3.2. Indirect Kalman Filter

Mainly due to measurement noises, *r̂*_1_, *υ̂*_1_ and *q̂*_1_ (position, velocity and attitude estimates of the right foot) have some errors. These errors are estimated using a Kalman filter. This kind of Kalman filters is called an indirect Kalman filter since errors in *r̂*_1_, *υ̂*_1_ and *q̂*_1_ are estimated instead of directly estimating *r*_1_, *υ*_1_ and *q*_1_.

Let *r_e,_*_1_, *υ_e,_*_1_, *q_e,_*_1_ and *b_e,_*_1_ be errors in *r̂*_1_, *υ̂*_1_, *q̂*_1_ and *b̂_g,_*_1_, which are defined by

(8) $$\begin{array}{lll} r_{e,1} & = & r_{1}-{\hat{r}}_{1}\\ \upsilon_{e,1} & = & \upsilon_{1}-{\hat{\upsilon }}_{1}\\ q_{e,1} & = & {\hat{q}}_{1}^{*}⊗q_{1}\\ b_{e,1} & = & b_{g,1}-{\hat{b}}_{g,1} \end{array}$$

where ⊗ is the quaternion multiplication. For a quaternion *q*, q* denotes the quaternion conjugate of *q*. Assuming the attitude error is small, *q_e,_*_1_ can be approximated as follows:

(9) $$q_{e,1}=\left[\begin{array}{c} 1\\ {\bar{q}}_{e,1} \end{array}\right]\in \left[\begin{array}{c} R\\ R^{3} \end{array}\right]$$

With this assumption, the attitude error can be represented by the three dimensional vector *q̄_e,_*_1_.

The multiplicative attitude error term *q_e,_*_1_ in Equation (8) is commonly used in the attitude estimation [16]. If we express the last Equation of (8) in the rotation matrix with the assumption (9), we have the following:

(10) $$\begin{array}{lll} C(q) & = & C(q_{e,1})C({\hat{q}}_{1})\\ & = & \left(I-2[{\hat{q}}_{e}\times ])C({\hat{q}}_{1}\right) \end{array}$$

For the left foot, *r_e,_*_2_, *υ_e,_*_2_, *q_e,_*_2_ and *b_e,_*_2_ can be defined similarly. If we combine the left and right foot variables, the state of a Kalman filter is defined by

(11) $$x=\left[\begin{array}{l} {\bar{q}}_{e,1}\\ r_{e,1}\\ \upsilon_{e,1}\\ {\bar{q}}_{e,2}\\ r_{e,2}\\ \upsilon_{e,2}\\ b_{e,1}\\ b_{e,2} \end{array}\right]\in \left[\begin{array}{l} R^{3}\\ R^{3}\\ R^{3}\\ R^{3}\\ R^{3}\\ R^{3}\\ R^{3}\\ R^{3} \end{array}\right]$$

The state space equation for one foot is a standard inertial navigation algorithm and is given in [9]. The state space equation for two feet is just a combination and is given by

(12) $$\dot{x}(t)=A(t)x(t)+w(t)$$

Where

$$A=\left[\begin{array}{cccccccc} \left[-(y_{g,1}-{\hat{b}}_{g,1})\times \right] & 0 & 0 & 0 & 0 & 0 & -0.5I & 0\\ 0 & 0 & I & 0 & 0 & 0 & 0 & 0\\ -2C\prime ({\hat{q}}_{1})[y_{a,1}\times ] & 0 & 0 & 0 & 0 & 0 & 0 & 0\\ 0 & 0 & 0 & \left[-(y_{g,2}-{\hat{b}}_{g,2})\times \right] & 0 & 0 & 0 & -0.5I\\ 0 & 0 & 0 & 0 & 0 & I & 0 & 0\\ 0 & 0 & 0 & -2C\prime ({\hat{q}}_{2})[y_{a,2}\times ] & 0 & 0 & 0 & 0\\ 0 & 0 & 0 & 0 & 0 & 0 & 0 & 0\\ 0 & 0 & 0 & 0 & 0 & 0 & 0 & 0 \end{array}\right]\;,\;w=\left[\begin{array}{c} -0.5\upsilon_{g,1}\\ 0\\ -C\prime ({\hat{q}}_{1})\upsilon_{a,1}\\ -0.5\upsilon_{g,2}\\ 0\\ -C\prime ({\hat{q}}_{2})\upsilon_{a,2}\\ \upsilon_{b,1}\\ \upsilon_{b,2} \end{array}\right]$$

Noises *υ_b,_*_1_ and *υ_b,_*_2_ are introduced to represent a slow change in the bias terms. In the definition of *A*, the symbol [*p*×] for a vector *p* = [*p*_1_ *p*_2_ *p*_3_]′ ∈ *R*^3^ is defined by

$$[p\times ]≜\left[\begin{array}{ccc} 0 & -p_{3} & p_{2}\\ p_{3} & 0 & -p_{1}\\ -p_{2} & p_{1} & 0 \end{array}\right]$$

There are two measurement equations for the state *x*(*t*). One is from vision data (Section 3.3). The other measurement equation (Section 3.4) is derived using the fact that the velocity of a foot is zero and *z* axis values are the same while a foot is on the flat floor.

### 3.3. Measurement Equation from the Vision Data

This section explains how the vision data is used in the Kalman filter.

**Figure 3.**
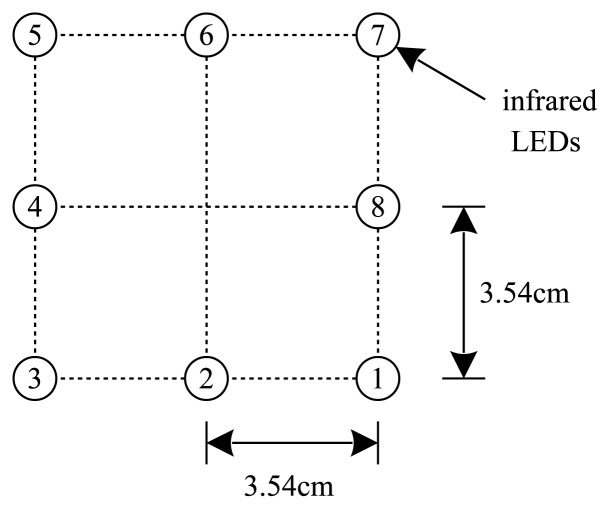
Eight infrared LED configuration.

There are eight infrared LEDs on the left foot as in Figure 3. A number is assigned to each LED. These LEDs are captured using the camera on the right foot. To simplify the image processing algorithm, an infrared filter is placed in front of the camera.

**Figure 4.**
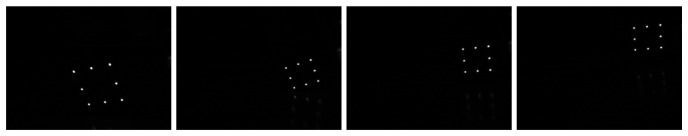
Infrared LED images during walking.

The typical infrared LED images during walking are given in Figure 4. A simple image processing algorithm can be used to obtain the center points of infrared LEDs.

Let the coordinates of the LEDs in the LED coordinate system be [led*_i_*]*_l_* ∈ *R*^3^ (1 ≤ *i* ≤ 8). Let [*u_i_ υ_i_*]′ ∈ *R*^2^ be the image coordinates of eight LEDs on the normalized image plane, which are obtained by applying the camera calibration parameters [17] to the pixel coordinates of eight LEDs. [led*_i_*]*_l_* and [*υ_i_ υ_i_*]′ satisfy the following relationship:

(13) $$s_{i}\;[\begin{array}{c} u_{i}\\ \upsilon_{i}\\ 1 \end{array}]=C_{l}^{c}{\left[{\text{led}}_{i}\right]}_{l}+{\left[\rho_{l}\right]}_{c}$$

where *s_i_* is the scaling factor. It is known that *ρ_l_*, $C_{l}^{c}$ and *s_i_* can be computed if the number of LEDs is equal to or more than four. We used the algorithm in [18] to compute *ρ_l_*, $C_{l}^{c}$ and *s_i_*. Only *ρ_l_* is used in the Kalman filter measurement equation.

To use Equation (13), we need to identify LED numbers from the LED image. In a general case where LEDs can rotate freely, it is impossible to uniquely identify the LED number. However, in our case, LEDs are attached on a shoe and the rotation is rather limited due to the mechanical structure of ankles. Thus it is not difficult to identify LED numbers from the images in Figure 4.

Let the estimated value of *ρ_l_* from the algorithm in [18] be defined by *ρ̂_l_*:

(14) $$\rho_{l}={\hat{\rho }}_{l}+\upsilon_{\text{vision}}$$

where *υ_vision_* denotes the estimation error in *ρ̂_l_*.

Inserting Equations (8), (10) and (14) into Equation (3), we have

(15) $$\left({\hat{r}}_{1}+r_{e,1})-({\hat{r}}_{2}+r_{e,2})={\left((I-2[{\bar{q}}_{e,2}\times ]){\hat{C}}_{n}^{b_{2}}\right)}^{\prime }p_{l}-{\left((I-2[{\bar{q}}_{e,1})\times ]{\hat{C}}_{n}^{b_{1}}\right)}^{\prime }p_{c}-{\left((I-2[{\bar{q}}_{e,1}\times ])C_{n}^{b_{1}}\right)}^{\prime }{\hat{C}}_{n}^{b_{1}}({\hat{p}}_{l}+\upsilon_{\text{vision}}\right)$$

Assuming *q̄_e,_*_1_ and *υ_vision_* are small, we can ignore the product term in Equation (15):

(16) $${\hat{r}}_{1}-{\hat{r}}_{2}-{\hat{C}}_{b_{2}}^{n}p_{l}+{\hat{C}}_{b_{1}}^{n}p_{c}+{\hat{C}}_{b_{1}}^{n}{\hat{C}}_{c}^{b_{1}}{\hat{\rho }}_{l}=-2{\hat{C}}_{b_{2}}^{n}[p_{l}\times ]{\bar{q}}_{e,2}+2{\hat{C}}_{b_{1}}^{n}[p_{c}\times ]{\bar{q}}_{e,1}-{\hat{C}}_{b_{1}}^{n}{\hat{C}}_{c}^{b_{1}}\upsilon_{\text{vision}}+2{\hat{C}}_{b_{1}}^{n}[(C_{c}^{b_{1}}{\hat{\rho }}_{l})\times ]{\bar{q}}_{e,1}-r_{e,1}+r_{e,2}$$

The left hand side of Equation (16) is denoted by *z_vision_* ∈ *R*^3^ and is used as a measurement equation in the Kalman filter:

$$z_{\text{vision}}≜{\hat{r}}_{1}-{\hat{r}}_{2}-{\hat{C}}_{b_{2}}^{n}p_{l}+{\hat{C}}_{b_{1}}^{n}p_{c}+{\hat{C}}_{b_{1}}^{n}C_{c}^{b_{1}}{\hat{\rho }}_{l}$$

In the matrix form, Equation (16) can be written as follows:

(17) $$z_{\text{vision}}=H_{\text{vision}}x+\upsilon_{\text{vision}}$$

where *υ_vision_* is the measurement noise and

$$H_{\text{vision}}=\left[\begin{array}{llllll} 2{\hat{C}}_{b_{1}}^{n}[(({\hat{C}}_{c}^{b_{1}}\hat{\rho })+p_{c})\times ] & -I & 0 & -2{\hat{C}}_{b_{2}}^{n}[p_{l}\times ] & I & 0 \end{array}\right]\in R^{3\times 18}$$

Whenever the camera on the right foot captures the LEDs on the left foot, Equation (17) can be used as a measurement equation.

### 3.4. Measurement Equations from Zero Velocity and Flat Floor Assumptions

During normal walking, a foot touches the floor almost periodically for a short interval. During this short interval, the velocity of a foot is zero and this interval is called a “zero velocity interval”.

The zero velocity interval is detected using accelerometers and gyroscopes [19]. In this paper, the detection method in [9] is used: the foot is assumed to be in the zero velocity interval if the change of the accelerometer is small and gyroscope values are small. The zero velocity intervals are detected separately for the left and right foot.

We assume that a person is walking on a flat floor. Thus, the *z* axis value of a foot in the navigation coordinate returns to a constant during the zero velocity interval (when a foot is on the floor). Using both zero velocity intervals and the flat floor assumptions, the measurement equation for the zero velocity interval of the right foot is given by

(18) $$\left[\begin{array}{c} 0-{\hat{\upsilon }}_{1}\\ z_{1,\text{floor}}-\left[\begin{array}{ccc} 0 & 0 & 1 \end{array}\right]\;{\hat{r}}_{1} \end{array}\right]=H_{1}x+\upsilon_{\text{zero},1}$$

where *z*_1,_*_floor_* is the *z* axis value when the right foot is on the floor and

$$H_{1}≜\left[\begin{array}{cccccccc} 0_{3\times 3} & 0_{3\times 3} & I_{3\times 3} & 0_{3\times 3} & 0_{3\times 3} & 0_{3\times 3} & 0_{3\times 3} & 0_{3\times 3}\\ 0_{1\times 3} & \left[\begin{array}{ccc} 0 & 0 & 1 \end{array}\right] & 0_{1\times 3} & 0_{1\times 3} & 0_{1\times 3} & 0_{1\times 3} & 0_{1\times 3} & 0_{1\times 3} \end{array}\right]$$

The measurement equation for the zero velocity interval of the left foot is given by

(19) $$\left[\begin{array}{c} 0-{\hat{\upsilon }}_{2}\\ z_{2,\text{floor}}-\left[\begin{array}{ccc} 0 & 0 & 1 \end{array}\right]\;{\hat{r}}_{2} \end{array}\right]=H_{2}x+\upsilon_{\text{zero},2}$$

where *z*_2,_*_floor_* is defined similarly with *z*_1,_*_floor_* and

$$H_{2}≜\left[\begin{array}{cccccccc} 0_{3\times 3} & 0_{3\times 3} & 0_{3\times 3} & 0_{3\times 3} & 0_{3\times 3} & I_{3\times 3} & 0_{3\times 3} & 0_{3\times 3}\\ 0_{1\times 3} & 0_{1\times 3} & 0_{1\times 3} & 0_{1\times 3} & \left[\begin{array}{ccc} 0 & 0 & 1 \end{array}\right] & 0_{1\times 3} & 0_{1\times 3} & 0_{1\times 3} \end{array}\right]$$

### 3.5. Kalman Filter Implementation

Here the implementation of the indirect Kalman filter is briefly explained. Detailed explanation for a similar problem can be found in [20]. All computations are done in the discrete time with the sampling period *T* = 0.01 second. The discrete time index *k* is used as usual; for example, discrete time value *r*_1_,*_k_* denotes the sampled value of continuous time value *r*_1_(*kT*).

The procedure to estimate *q*_1,_*_k_*, *υ*_1,_*_k_*, *r*_1,_*_k_*, *q*_2,_*_k_*, *υ*_2,_*_k_* and *r*_2,_*_k_* is as follows:

- *q̂*_1,_*_k_*, *υ̂*_1,_*_k_*, *r̂*_1,_*_k_*, *q̂*_2,_*_k_*, *υ̂*_2,_*_k_* and *r̂*_1,_*_k_* are computed using the discretized Equation of (6).
- The time update step [21] of the Kalman filter using Equation (12) is performed.
- The measurement update step using Equations (17)–(19) is performed to compute *x̂*.
- Using *x̂*, *q̂*_1,_*_k_*, *υ̂*_1,_*_k_*,*r̂*_1,_*_k_* and *b̂_g,_*_1_ are updated as follows:
  $$\begin{array}{ll} {\hat{r}}_{1,k} & ={\hat{r}}_{1,k}+{\hat{r}}_{e,1,k}\\ {\hat{\upsilon }}_{1,k} & ={\hat{\upsilon }}_{1,k}+{\hat{\upsilon }}_{e,1,k}\\ {\hat{b}}_{g,1,k} & ={\hat{b}}_{g,1,k}+{\hat{b}}_{e,1,k}\\ {\hat{q}}_{1,k} & ={\hat{q}}_{1,k}⊗{\hat{q}}_{e,1,k} \end{array}$$
- Similarly, *q̂*_2,_*_k_*, *υ̂*_2,_*_k_*, *r̂*_2,_*_k_* and *b̂_g,_*_2_ are updated.
- After the update, *x̂* is set to a zero vector.
- The discrete time index *k* is increased and the procedure is repeated.

## 4. Smoother

**Figure 5.**
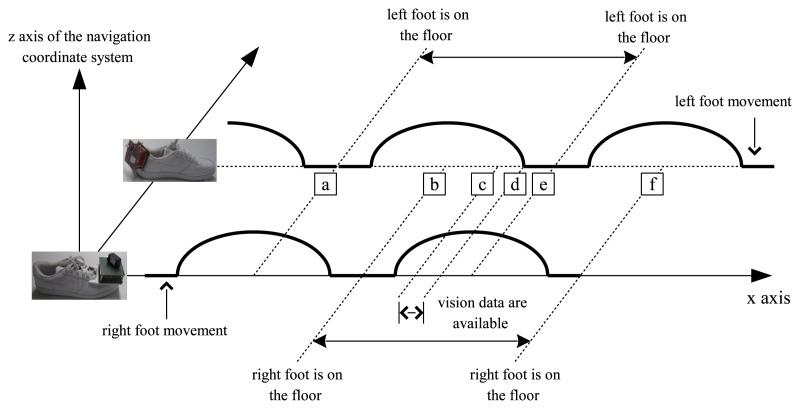
Typical two feet movement in the navigation coordinate system.

In Figure 5, typical two feet movement during walking is illustrated in the navigation coordinate system. Suppose the right foot is on the floor in the area around (b). As the right foot is taking off the floor ((b)–(d) area), the left foot is touching on the floor in the area around (e). From the configuration of the camera, LED images are available in the (c)–(d) interval.

For the left foot, the measurement data are available in the area around (a) (zero velocity update) and (c)–(d) (vision data update). When the measurement data are not available, the motion estimation depends on double integration of acceleration, whose errors tend to increase quickly even for a short time. To get a smooth motion trajectory, a forward-backward smoother (Section 8.5 in [21]) is applied.

A smoother algorithm is applied for each walking step separately on the left and right foot movement. For example, consider the left foot movement between (a) and (e). After computing the forward Kalman filter (that is, a filter in Section 3.2) up to the point (e), the backward Kalman filter is computed from (e) to (a) with the final value of the forward Kalman filter as an initial value. Since the final value of the forward filter is used in the backward filter, the forward and the backward filter become correlated. Thus the smoother is not optimal. However, we found that the smoothed output is good enough for our application.

Note that *r̂*_2,_*_k_* is the position of the left foot, which is computed by the forward filter in Section 3.2. Let *r̂*_2,_*_b,__k_* be the position of the left foot, which is computed by a backward Kalman filter. Two values *r̂*_2,_*_k_* and *r̂*_2,_*_b,__k_* are combined using simple weighting functions *w*_2,_*_f,__k_* and *w*_2,_*_b,__k_* as follows:

(20) $${\hat{r}}_{2,s,k}=\frac{w_{2,b,k}}{w_{2,f,k}+w_{2,b,k}}{\hat{r}}_{2,k}+\frac{w_{2,f,k}}{w_{2,f,k}+w_{2,b,k}}{\hat{r}}_{2,b,k}$$

The weighting functions *w*_2,_*_f,__k_* and *w*_2,_*_b,__f_* are given by

(21) $$\begin{array}{lll} w_{2,f,k} & = & \alpha \beta^{k-M_{1}}\\ w_{2,b,k} & = & \alpha \beta^{M_{2}-k_{1}},\;\beta >1 \end{array}$$

where the discrete time indices of one walking step is assumed to be [*M*_1,_ *M*_2_]. Consider one walking step from (a) to (e) in Figure 5. With the weighting functions in Equation (21), *r̂*_2,_*_s,k_* ≈ *r̂*_2,_*_k_* near the position (a) (that is, near the discrete time *M*_1_) and *r̂*_2,_*_s,__k_* ≈ *r̂*_2,_*_b,__k_* near the position (e). Thus the weighting functions in Equation (21) provide a simple way to combine the forward and backward filters.

A smoother algorithm can be applied to the velocity and attitude similarly.

## 5. Experiments

**Figure 6.**
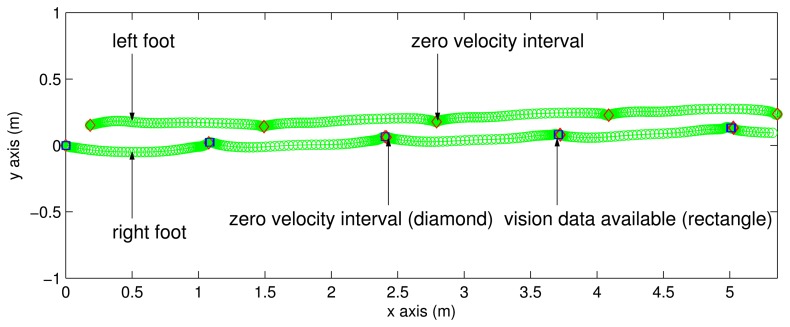
Estimated two feet trajectories on the *xy* plane.

To verify the proposed system, a person walked on the floor and the two feet motion was estimated using the proposed algorithm. The estimated two feet trajectory on the *xy* plane in the navigation coordinate system is given in Figure 6. Since the *x* direction of the navigation coordinate system can be chosen arbitrarily, the trajectories are rotated so that the walking direction coincides with the *x* axis. The left foot trajectory is the upper one and the right foot trajectory is the lower one. The zero velocity intervals are indicated with the diamond symbols. The rectangle symbols indicate that vision data are available at those positions (that is, LEDs on the left foot can be seen from the camera on the right foot).

**Figure 7.**
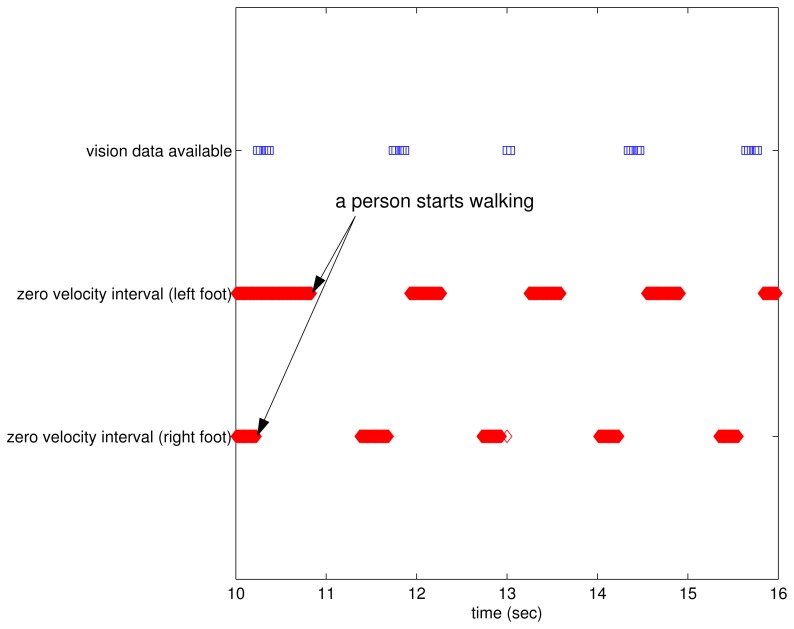
Zero velocity intervals and vision data available intervals.

In the time domain, the relationship between zero velocity intervals and vision data available intervals is given in Figure 7. As illustrated in Figure 5, vision data are available between the right foot zero velocity intervals and the left foot zero velocity intervals during walking.

**Figure 8.**
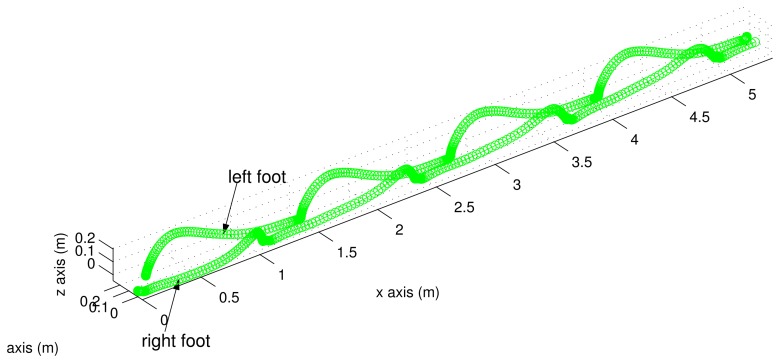
Estimated trajectories in the three dimensional space.

Three dimensional trajectories are given in Figure 8. There is a difference between the left and right foot motion patterns. This is due to the difference in the positions of inertial sensors: the inertial sensor unit is on the front in the case of the right foot and on the back in the case of the left foot (see Figure 1).

**Figure 9.**
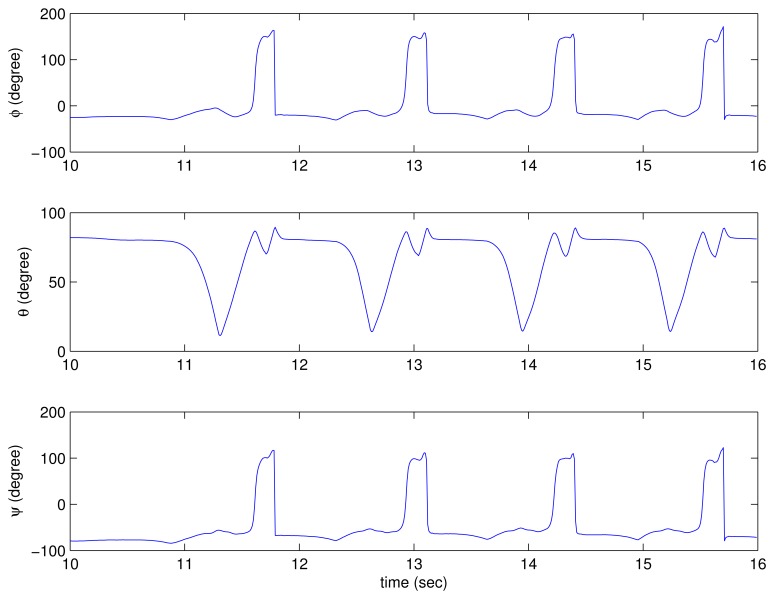
Estimated attitude of the left foot (Euler angles).

In addition to trajectories, attitude and velocity are also available from the inertial navigation algorithm. For example, estimated attitude (in Euler angles) of the left foot is given in Figure 9.

Thus we can obtain key gait analysis parameters such as step length, stride length, foot angle and walking speed using the proposed system.

**Figure 10.**
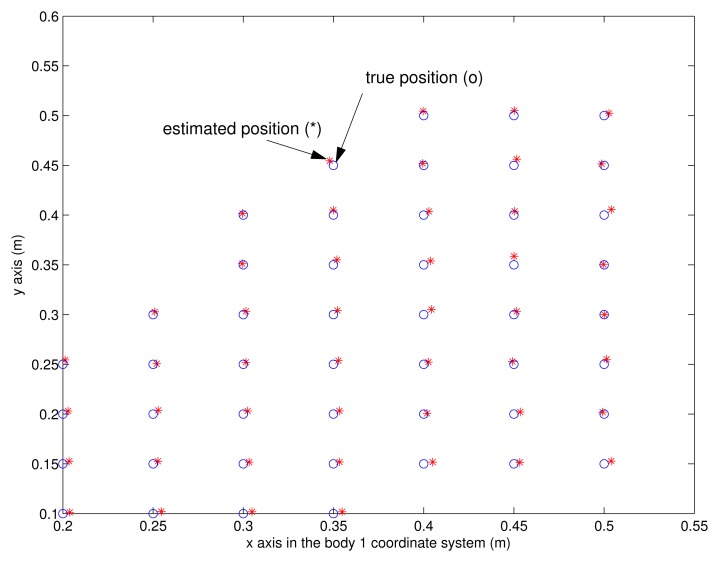
Vision-based position estimation accuracy experiment results in the body 1 coordinate system.

Now the accuracy of the proposed system is evaluated. First, we test the accuracy of the vision-based position estimation, which is used to estimate the vector between two feet. The left shoe is located on different positions of the grid while the right shoe is located on the fixed position. The estimated left shoe position with respect to the right shoe is compared with the true value, which can be obtained from the grid. The result is given in Figure 10, where the position represents the origin of the body 2 coordinate system in the body 1 coordinate system. We can see the position can be accurately estimated using the proposed system (eight infrared LEDs). The mean error distance is 0.4 cm and the maximum error distance is 0.8 cm.

**Figure 11.**
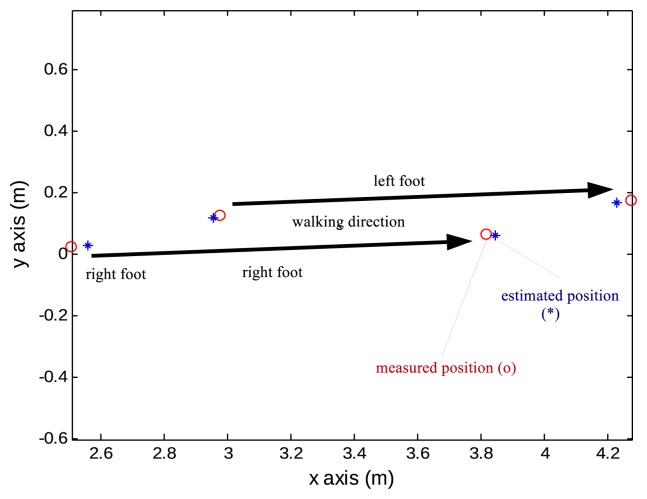
One walking step estimation accuracy.

The next task is to evaluate the accuracy of the trajectories. A person walked on the long white paper with marker pens attached on both shoes. Marker pens are attached on shoes so that dots are marked on the white paper whenever a foot touches the floor. Marked dot positions are measured with a ruler and these values are considered as true values. The estimation positions during zero velocity intervals (when one foot is on the floor) are compared with marked dots. One step result is given in Figure 11.

**Figure 12.**
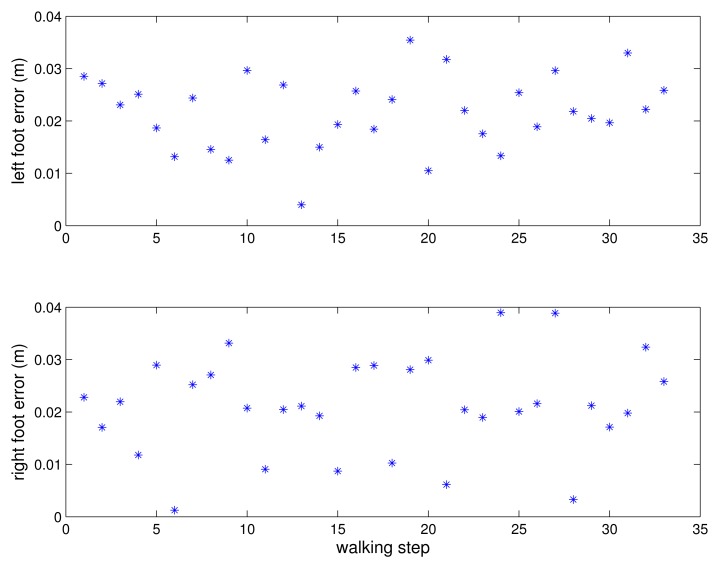
Step length estimation error.

**Figure 13.**
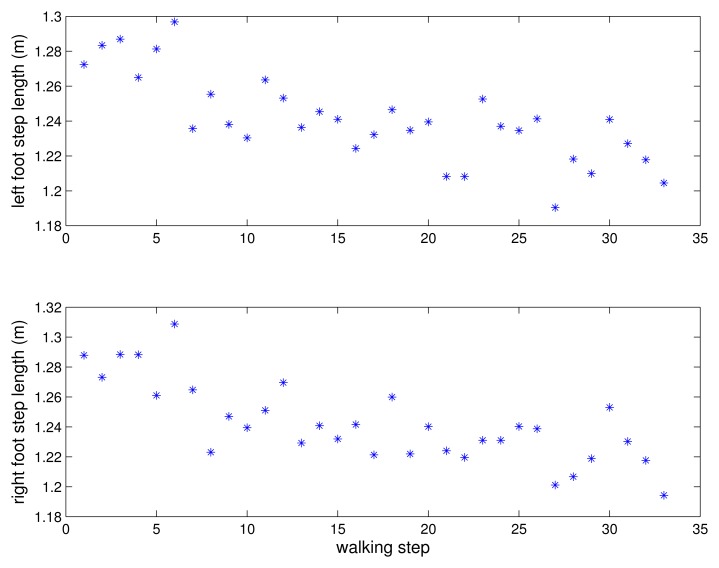
Estimated Step length.

A person walked 33 steps and the errors between the estimated positions and the marked positions are given in Figure 12 for each step. The estimated step length is given in Figure 13. The mean errors are 2.2 cm for the left foot and 2.1 cm for the right foot. The maximum errors are 3.6 cm for the left foot and 3.89 cm for the right foot. Two more experiments were done and the mean errors are 2.5 cm and 1.2 cm for the left foot and 2.5 cm and 1.7 cm for the right foot. The maximum errors are 4.1 cm and 2.8 cm for the left foot and 3.9 cm and 5.4 cm for the right foot. The 2 cm level error is too large for the kinetic calculations. However, the proposed system is suitable for the gait analysis system requiring basic gait parameters such as walking step length and walking speed.

## 6. Conclusions

Using inertial sensors on shoes, two feet motion is estimated using an inertial navigation algorithm. When two feet motion is estimated, it is necessary to measure the relative position between the two feet. In the proposed system, a vision system is used to measure the relative position and attitude between two feet.

Using the proposed system, we can obtain quantitative gait analysis parameters such as step length, stride length, foot angle and walking speed. Also we can see three dimensional trajectories of the two feet, which give qualitative information for gait analysis.

The accuracy of the proposed system is evaluated by measuring the position of a foot when a foot touches the floor. The mean position error is 1.2–2.5 cm and the maximum position error is 5.4 cm. For gait analysis, we believe the error is in an acceptable range.

The main contribution of the proposed system is that two feet motion can be observed at any place as long as the floor is flat. In commercial motion tracking using a camera such as Vicon, a dedicated experiment space is required. Thus we believe natural walking patterns can be observed using the proposed system.
